# Evidence for Hypoxia-Induced Shift in ATP Production from Glycolysis to Mitochondrial Respiration in Pulmonary Artery Smooth Muscle Cells in Pulmonary Arterial Hypertension

**DOI:** 10.3390/jcm12155028

**Published:** 2023-07-31

**Authors:** Satoshi Akagi, Kazufumi Nakamura, Megumi Kondo, Satoshi Hirohata, Heiichiro Udono, Mikako Nishida, Yukihiro Saito, Masashi Yoshida, Toru Miyoshi, Hiroshi Ito

**Affiliations:** 1 Department of Cardiovascular Medicine, Faculty of Medicine, Dentistry and Pharmaceutical Sciences, Okayama University, Okayama 700-8558, Japan; ichibun@cc.okayama-u.ac.jp (K.N.); p9wu565f@okayama-u.ac.jp (M.K.); p5438a3l@s.okayama-u.ac.jp (Y.S.); yoshid-m@cc.okayama-u.ac.jp (M.Y.); miyoshit@cc.okayama-u.ac.jp (T.M.); itomd1602@gmail.com (H.I.); 2 Department of Medical Technology, Graduate School of Health Sciences, Okayama University, Okayama 700-8558, Japan; hirohas@cc.okayama-u.ac.jp; 3 Department of Immunology, Graduate School of Medicine, Dentistry, and Pharmaceutical Sciences, Okayama University, Okayama 700-8558, Japan; udono@cc.okayama-u.ac.jp (H.U.); pzd18dj7@s.okayama-u.ac.jp (M.N.)

**Keywords:** glycolysis, mitochondrial respiration, pulmonary arterial hypertension, pulmonary artery smooth muscle cells, Seahorse technology, hypoxia, ATP production

## Abstract

Background: The metabolic state of pulmonary artery smooth muscle cells (PASMCs) from patients with pulmonary arterial hypertension (PAH) is not well understood. In this study, we examined the balance between glycolysis and mitochondrial respiration in non-PAH-PASMCs and PAH-PASMCs under normoxia and hypoxia. Methods: We investigated the enzymes involved in glycolysis and mitochondrial respiration, and studied the two major energy-yielding pathways (glycolysis and mitochondrial respiration) by measuring extracellular acidification rate (ECAR) and cellular oxygen consumption rate (OCR) using the Seahorse extracellular flux technology. Results: Under both normoxia and hypoxia, the mRNA and protein levels of pyruvate dehydrogenase kinase 1 and pyruvate dehydrogenase were increased in PAH-PASMCs compared with non-PAH-PASMCs. The mRNA and protein levels of lactate dehydrogenase, as well as the intracellular lactate concentration, were also increased in PAH-PASMCs compared with non-PAH-PASMCs under normoxia. However, these were not significantly increased in PAH-PASMCs compared with non-PAH-PASMCs under hypoxia. Under normoxia, ATP production was significantly lower in PAH-PASMCs (59 ± 5 pmol/min) than in non-PAH-PASMCs (70 ± 10 pmol/min). On the other hand, ATP production was significantly higher in PAH-PASMCs (31 ± 5 pmol/min) than in non-PAH-PASMCs (14 ± 3 pmol/min) under hypoxia. Conclusions: There is an underlying change in the metabolic strategy to generate ATP production under the challenge of hypoxia.

## 1. Introduction

Pulmonary arterial hypertension (PAH) is a life-threatening disease characterized by abnormally elevated pulmonary arterial pressure, which can lead to heart failure [1]. PAH has several underlying pathological mechanisms such as endothelial dysfunction, epithelial–mesenchymal transition, apoptosis resistance, and higher proliferation in pulmonary artery endothelial cells and pulmonary artery smooth muscle cells (PASMCs), leading to vascular remodeling and PAH development [2]. This is known as the cancer theory of PAH [3,4,5].

Cancer cells show recognizable hallmarks in their energy metabolism, as initially proposed by Otto Warburg [6]. He observed that cancerous tissue generated large amounts of lactate even under normoxia compared with normal tissues. This is known as the Warburg effect or aerobic glycolysis [6]. Warburg proposed that cancer cells cause an injury to respiration as they progress from a differentiated cellular state to a proliferative state [6]. On the other hand, the majority of cancer cells use respiration to drive increased flux through the tricarboxylic acid (TCA) cycle, which promotes tumor growth [7]. In cancer metabolism, TCA cycle metabolites sustain histone acetylation and inhibit DNA, RNA, and histone demethylases. Thus, cancer cells use metabolism to maintain their malignant phenotype and support tumor progression [8].

Various pathway such as glycolysis, mitochondrial respiration, pentose phosphate pathway, β oxidation, and glycogenesis are involved in energy metabolism. HIF-1α, Akt, AMPK, and ROS are associated with these pathways. Chronic hypoxia acts on the lung to cause HIF-dependent vascular remodeling, which leads to pulmonary hypertension [9]. Activated HIF-1α is found in cancer. Therefore, studying the hypoxia responses of PAH-PASMCs is important. PAH-PASMCs have been shown to increase glycolysis under normoxia [10]. However, the metabolic state of PAH-PASMCs depending on oxygen level is not well understood. In this study, we examine how glycolysis and mitochondrial respiration are balanced in both PAH-PASMCs and non-PAH-PASMCs under normoxia and hypoxia.

We examined the enzymes including pyruvate dehydrogenase kinase 1 (PDK1), pyruvate dehydrogenase E1 subunit alpha 1 (PDHA1), and lactate dehydrogenase A subunit (LDHA). PDK1 negatively regulates PDHA1 activity by phosphorylating PDHA1. PDHA1 catalyzes the conversion of pyruvate to acetyl-CoA along with acetyl-CoA from the fatty acid β oxidation. We also used the Seahorse technology to investigate the major ATP-yielding pathways (glycolysis and mitochondrial respiration). We measured the cellular oxygen consumption rate (OCR) to examine mitochondrial respiration and the extracellular acidification rate (ECAR) to examine the glycolysis for both non-PAH-PASMCs and PAH-PASMCs under normoxia and hypoxia.

## 2. Materials and Methods

### 2.1. Isolation of Human Pulmonary Artery Smooth Muscle Cells

For PAH-PASMC isolation, pulmonary arteries were obtained from six patients with PAH (four men and two women) during lung transplantations (Table 1). Types of PAH examined included five patients with idiopathic PAH and one patient with PAH associated with congenital heart diseases. For non-PAH experiments, non-PAH-PASMCs were obtained from three patients (two men and one woman). These samples were obtained from patients with lung cancer at lung lobectomy. All human subject protocols were approved by the Human Ethics Committee of the Okayama University Graduate School of Medicine, Dentistry, and Pharmaceutical Sciences (protocol code 1511-017), and written informed consent was obtained from all patients before the procedure. This study complies with the Declaration of Helsinki and was performed according to ethics committee approval (protocol code 1511-017). In brief, peripheral pulmonary arteries smaller than 1 mm in outer diameter were disaggregated with collagenase in a water bath for 15 min at 37 °C. The adventitia and intima were removed, and isolated arteries were cut into sections 2 mm in length. The cut arteries were placed in a 6-well plate with Dulbecco’s modified Eagle’s medium (DMEM; Gibco, Grand Island, NY, USA) supplemented with 10% fetal bovine serum (FBS; Sigma-Aldrich, St. Louis, MO, USA) and 0.1 mg/mL kanamycin (Sigma-Aldrich). They were incubated in a humidified 5% CO_2_ atmosphere at 37 °C. We used separated cells isolated from individuals. Isolated cells were identified by positive staining with antibodies against α-smooth muscle actin, myosin, and smoothelin [11]. Cells between passages 3 and 5 were used for all experiments. PASMCs were seeded in 24-well plates at a density of 5 × 10^4^ cells/well.

### 2.2. Exposure to Normoxia and Hypoxia

Non-PAH-PASMCs and PAH-PASMCs were cultured under normal oxygen tension (20% O_2_, 5% CO_2_) or exposed to hypoxia (2% O_2_, 5% CO_2_, 92% N_2_) for 72 h. Dichloroacetic acid (DCA: 347795, Sigma-Aldrich) or vehicle were administered to PAH-PASMCs under either normoxia or hypoxia for 72 h.

### 2.3. Quantitative RT-PCR Analysis

Total RNA was extracted from non-PAH-PASMCs and PAH-PASMCs using the TaKaRa NucleoSpin^®^ RNA kit (Takara Bio, Ohtsu, Japan) according to the manufacturer’s instructions. The total RNA (2 μg) from each sample was used to generate complementary DNA with ReverTra Ace (TOYOBO, Osaka, Japan). Quantitative real-time PCR was performed using the Applied Biosystems 7300 Real-Time PCR System (Applied Biosystems). The PCR primers specific to the following genes were used: *PDK1* (Hs_PDK1_1_SG QuantiTect Primer Assay (Table 2); NM_002610; Qiagen); *PDH* (Hs_PDHA1_1_SG QuantiTect Primer Assay; NM_000284; Qiagen); *LDH* (Hs_LDHA_1_SG QuantiTect Primer Assay; NM_001135239; Qiagen); and *ACTB* (Mm_Actb_1_SG QuantiTect Primer Assay; NM_007393). The annealing temperature was 60 °C. The data were analyzed using the 2-ΔΔct method.

### 2.4. Western Blot Analysis

Western blotting was performed as previously described [12]. Briefly, total cell lysates of non-PAH-PASMCs and PAH-PASMCs were extracted in commonly used radioimmunoprecipitation buffer with 10 µg/mL phenylmethylsulfonyl fluoride (Sigma) and then concentrated by centrifugation at 12,000 rpm for 20 min. Protein samples (7 µg) were loaded on 10% sodium dodecyl sulfate-polyacrylamide gel and blotted onto nitrocellulose membranes. Blots were incubated with anti-PDK1 antibody (1:1000; #3820; Cell Signaling Technology, Danvers, MA, USA), anti-PDH phospho S293 (1:1000; ab177461, Abcam, Cambridge, UK), anti-PDH (1:1000; ab110330, Abcam), and anti-beta actin (1:1000; ab6276, Abcam). The secondary antibody was horseradish-peroxidase-conjugated anti-mouse (1:10,000; NA931, GE Healthcare, Chicago, IL, USA) or anti-rabbit (1:10,000; NA934, GE Healthcare) IgG antibody. Positive signals were detected by a chemiluminescence system (ECL plus, GE Healthcare Bio-Sciences, Piscataway, NJ, USA).

### 2.5. Lactate Assay

Intracellular lactate was measured by Lactate Colorimetric/Fluorometric assay kit (Catalog # K607, BioVision, Boston, MA, USA) according to the manufacturer’s instructions. The cells were resuspended in assay buffer and homogenized on ice. The lysate was then centrifuged and the supernatant was collected. The supernatant was then loaded on to a 10 kDa spin column for deproteinization.

### 2.6. Energy Metabolism Analysis

OCR and ECAR were measured using the XF Cell Mito Stress Test and XF Glycolysis Stress test, respectively, on an Extracellular Flux Analyzer XFp (Agilent Technologies, Santa Clara, CA, USA). ATP production was measured by calculating the change in OCR using Cell Mito Stress test: (last rate measurement before Oligomycin injection)—(Minimum rate measurement after Oligomycin injection). PASMCs were seeded in 96-well plates at a density of 2 × 10^4^ cells/well with DMEM medium supplemented with 10% FBS and 0.1 mg/mL kanamycin. Agilent Seahorse XF Base Medium with glucose (final concentration: 10 mM), sodium Pyruvate (final concentration: 1 mM), and L-glutamine (final concentration: 2 mM) were used for the analysis medium. Oligomycin (final concentration: 2.0 μM), FCCP (final concentration: 2.0 μM), and Rotenone antimycin A (final concentration: 0.5 μM) were for performing Cell Mito Stress Test. Glucose (final concentration: 10 mM), Oligomycin (final concentration: 1.0 μM), and 2-DG (final concentration: 50 mM) were for performing Glycolysis Stress test. We incubated the cartridge with a O_2_-free and CO_2_-free atmosphere at 37 °C to avoid the risk of cell reoxygenation during the Seahorse analysis.

### 2.7. Transmission Electron Microscopy

Transmission Electron Microscopy of non-PAH-PASMCs and PAH-PASMCs was performed by Tokai Electron Microscopy, Inc. Digital images were taken with CCD camera. We observed digital images of 50 mitochondria in both PAH-PASMCs and non-PAH-PASMCs under normoxia or hypoxia. We compared morphologies (outer membrane, inter membrane space, inner membrane, matrix).

### 2.8. Animal Study Protocol

This investigation conforms to the Guide for the Care and Use of Laboratory Animals published by the US National Institutes of Health (NIH Publication No. 85-23, revised 1985). The animal protocol was approved by the Animal Ethics Committee of Okayama University Graduate School of Medicine, Dentistry, and Pharmaceutical Sciences (Permit number: OKU-2022579). This study was carried out in compliance with the ARRIVE guidelines. Sugen–hypoxia–normoxia (SuHx) PAH rat models were used. Adult male Wister rats (Charles River, Yokohama, Japan; 200 to 250 g in body weight) received a single subcutaneous injection of SU5416 (20 mg/kg; Cayman Chemical, Ann Arbor, MI, USA) and were exposed to hypoxia (10% O_2_) or normoxia for 3 weeks. The animals were assigned to a group that either received vehicle or DCA (80 mg/kg/day) for three weeks after SU5416 injection. For the group kept under normoxic conditions, hemodynamics and lung tissues were examined 2 weeks following the injection. All surgery was performed under inhalation of isoflurane (1.8% *v/v*) anesthesia, and all efforts were made to minimize suffering. The rats were anesthetized with inhalation of isoflurane, and a high-fidelity 1.4F Millar catheter (Millar Instruments Inc., Houston, TX, USA) was inserted into the right ventricle via the right jugular vein. Right ventricular (RV) systolic pressure was measured by the PowerLab System with the use of Chart 5.0 software. After hemodynamics had been recorded, the rats were euthanized and their lungs and heart were isolated. The RV wall was dissected from the left ventricle (LV) and ventricular septum (S). The wet weight of the RV and LV + S was determined, and RV hypertrophy was expressed as RV weight/LV + S weight. For histological analysis, the lungs were fixed in 10% neutral buffered formalin. For paraffin embedding, the lungs were dissected in tissue blocks from all lobes; then, 3 μm sections were taken. To assess the type of remodeling in the muscular pulmonary arteries, Elastica van Gieson staining was performed according to common histopathological procedures. For each rat, 30 to 40 medial areas of intra-acinar arteries were measured and then averaged (the range of diameters was between 25 and 50 μm).

### 2.9. Statistical Analysis

Data are presented as means ± standard deviations. Differences between groups were assessed by analysis of variance and post-hoc Student’s *t* tests for multiple comparisons. A *p* value < 0.05 was considered significant. Statistical analyses were performed using SPSS version 25.0 (IBM Corp., Armonk, NY, USA).

## 3. Results

### 3.1. PDK1, PDHA1, and LDHA Expression and Intracellular Lactate Production under Normoxia and Hypoxia

**Figure 1:**
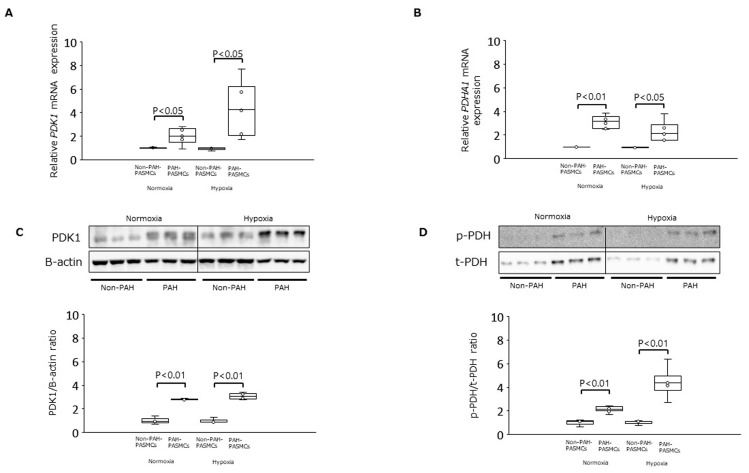
Expression of PDK-1 and PDHA1 in PAH-PASMCs and non-PAH-PASMCs under normoxia and hypoxia. (**A**) Relative levels of PDK1 mRNA in PAH-PASMCs (n = 6) and non-PAH-PASMCs (n = 3). (**B**) Relative levels of PDHA1 mRNA. (**C**) PDK1/B-actin ratio. (**D**) Phosphorylated PDH (p-PDH)/total PDH (t-PDH) ratio. PDK1: pyruvate dehydrogenase kinase 1; PDHA1: pyruvate dehydrogenase E1 subunit alpha 1; PAH: pulmonary arterial hypertension; PASMCs: pulmonary artery smooth muscle cells.

Under normoxic conditions, the levels of mRNA encoding PDK1 and PDHA1 were significantly increased in PAH-PASMCs compared with non-PAH-PASMCs by factors of 2.0-fold (*p* < 0.05) and 3.1-fold (*p* < 0.01), respectively (Figure 1A,B). The levels of PDK1 protein and PDH phosphorylation were also significantly increased in PAH-PASMCs by factors of 2.8-fold (*p* < 0.01) and 2.2-fold (*p* < 0.01), respectively, compared with non-PAH-PASMCs under normoxia (Figure 1C,D). Similar results were obtained under hypoxic conditions: the mRNA levels of PDK1 and PDHA1 were significantly increased in PAH-PASMCs compared with non-PAH-PASMCs by 4.3-fold (*p* < 0.05) and 2.3-fold (*p* < 0.05), respectively (Figure 1A,B). Consistently, the amounts of PDK1 protein and PDH phosphorylation were also significantly increased in PAH-PASMCs compared with non-PAH-PASMCs under hypoxia by 3.1-fold (*p* < 0.01) and 4.4-fold (*p* < 0.01), respectively (Figure 1C,D).

**Figure 2:**
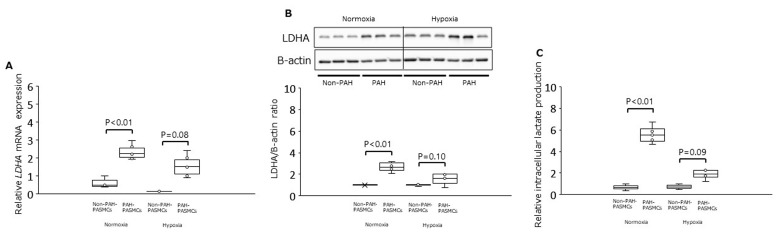
Expression of LDHA and intracellular lactate production in PAH-PASMCs and non-PAH-PASMCs under normoxia and hypoxia. (**A**) Relative levels of LDAH mRNA. (**B**) LDH/B-actin ratio. (**C**) Relative amounts of intracellular lactate. LDHA: lactate dehydrogenase A subunit.

Under normoxia, the mRNA level for LDHA was significantly increased in PAH-PASMCs by 2.4-fold compared with non-PAH-PASMCs (*p* < 0.01) (Figure 2A). The LDHA protein was significantly increased in PAH-PASMCs compared with non-PAH-PASMCs by 2.7-fold (*p* < 0.01) (Figure 2B). However, LDHA mRNA and LDHA protein were not significantly increased in PAH-PASMCs compared with non-PAH-PASMCs under hypoxic conditions by factors of 2.0-fold (*p* = 0.08) and 3.1-fold (*p* = 0.10), respectively (Figure 2A,B). Under normoxic conditions, the concentration of intracellular lactate was significantly increased in PAH-PASMCs compared with non-PAH-PASMCs by a factor of 3.4-fold (*p* < 0.01). However, the concentration of intracellular lactate was not significantly increased in PAH-PASMCs compared with non-PAH-PASMCs under hypoxia, consistent with LDHA mRNA levels (*p* = 0.09) (Figure 2C). These results suggest that PAH-PASMCs undergo a significant metabolic shift toward anaerobic glycolysis, even under normoxic conditions.

### 3.2. Energy Metabolism in Normoxia and Hypoxia

**Figure 3:**
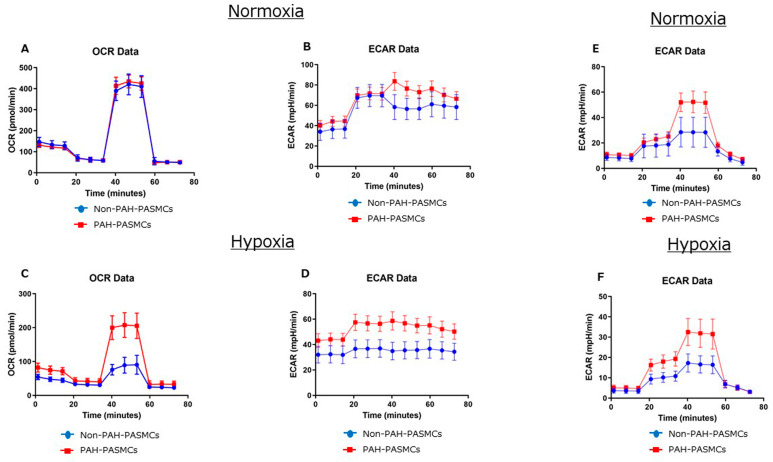
Representative Seahorse graphs from Cell Mito Stress test (**A**–**D**) and Glycolysis Stress test (**E**,**F**) for non-PAH-PASMC and PAH-PASMC under normoxia and hypoxia.

We next measured the OCR and ECAR for both non-PAH-PASMCs and PAH-PASMCs under normoxic and hypoxic conditions (Table 3). Representative Seahorse graphs from Cell Mito Stress test and Glycolysis Stress test for non-PAH-PASMC and PAH-PASMC are shown in Figure 3.

Basal OCR levels were significantly lower for PAH-PASMCs (70 ± 5 pmol/min) than for non-PAH-PASMCs (79 ± 4 pmol/min) (*p* < 0.01) under normoxic conditions. Conversely, under hypoxia, basal OCR levels were significantly higher for PAH-PASMCs (39 ± 4 pmol/min) than for non-PAH-PASMCs (22 ± 3 pmol/min) (*p* < 0.01). Basal ECAR levels were significantly higher in PAH-PASMCs (44 ± 3 mpH/min) than in non-PAH-PASMCs (37 ± 2 mpH/min) (*p* < 0.01) under normoxia. Similarly, under hypoxia, basal ECAR levels were significantly higher in PAH-PASMCs (44 ± 3 mpH/min) than in non-PAH-PASMCs (32 ± 3 mpH/min) (*p* < 0.01). ATP production was significantly lower in PAH-PASMCs (59 ± 2 pmol/min) than in non-PAH-PASMCs (70 ± 2 pmol/min) (*p* < 0.01) under normoxia. Conversely, ATP production was significantly higher in PAH-PASMCs (31 ± 3 pmol/min) than in non-PAH-PASMCs (14 ± 2 pmol/min) (*p* < 0.01) under hypoxic conditions. We examined the glycolytic functions using the Glycolysis Stress test. Glycolysis yield, glycolytic capacity, and glycolytic reserve were significantly higher in PAH-PASMCs than in non-PAH-PASMCs under both normoxia and hypoxia (Table 4).

These results show that glycolysis is facilitated in PAH-PASMCs under normoxia. Conversely, under hypoxic conditions, mitochondrial respiration is preserved in PAH-PASMCs compared with non-PAH-PASMCs under hypoxia, although hypoxia reduced mitochondrial respiration in both PAH-PASMCs and non-PAH-PASMCs.

### 3.3. Mitochondrial Morphology in Normoxia and Hypoxia

**Figure 4:**
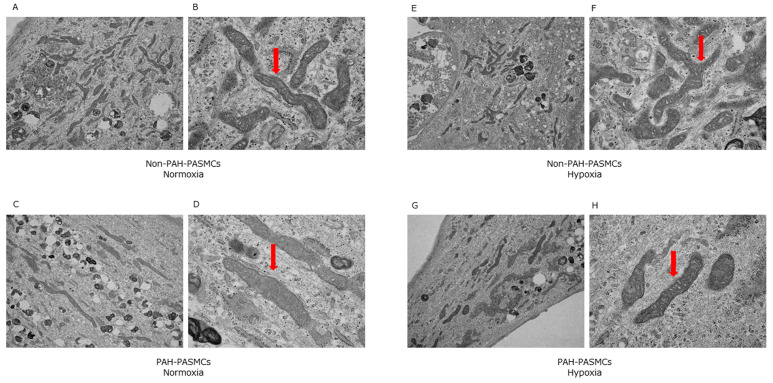
Transmission electron microscopy images of mitochondrial morphology under normoxic and hypoxic conditions. Arrows show mitochondria. Non-PAH-PASMCs under normoxia (**A**,**B**). PAH-PASMCs under normoxia (**C**,**D**). Non-PAH-PASMCs under hypoxia (**E**,**F**). PAH-PASMCs under hypoxia (**G**,**H**).

We next evaluated the mitochondrial morphology in both PAH-PASMCs and non-PAH-PASMCs under normoxia and hypoxia. We observed no significant differences in mitochondrial morphologies (Figure 4). These data suggest that the differences in energy metabolism between non-PAH-PASMCs and PAH-PASMCs under normoxia and hypoxia do not stem from differences in mitochondrial morphology.

### 3.4. Dichloroacetic Acid Did Not Increase ATP Production and Improve Pulmonary Hypertension

**Figure 5:**
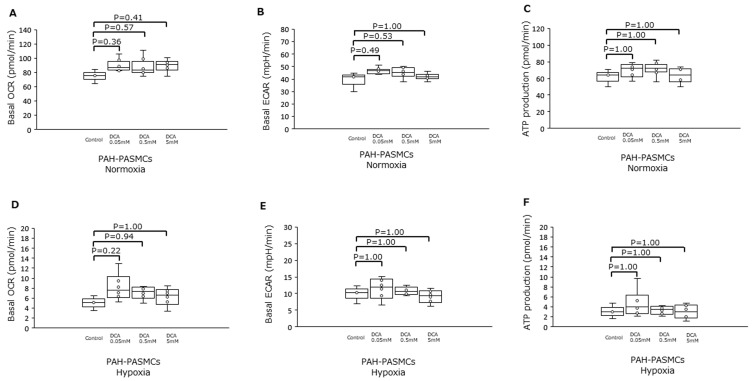
Effects of dichloroacetate on PAH-PASMCs under either normoxia or hypoxia. (**A**) Basal OCR under normoxia in the presence of increasing amounts of DCA. (**B**) Basal ECAR under the same conditions as in (**A**). (**C**) ATP production for the same conditions as in (**A**). (**D**) Basal OCR for cells under hypoxia with increasing amounts of DCA. (**E**) Basal ECAR under the same conditions as in (**D**). (**F**) ATP production under the same conditions as in (**D**). DCA: dichloroacetate.

We then assessed the effects of DCA, an inhibitor of the mitochondrial enzyme PDK1, in PAH-PASMCs under normoxia and hypoxia (Figure 5). There were no significant differences in basal OCR, ECAR, and ATP production between control and DCA under normoxia and hypoxia.

**Figure 6:**
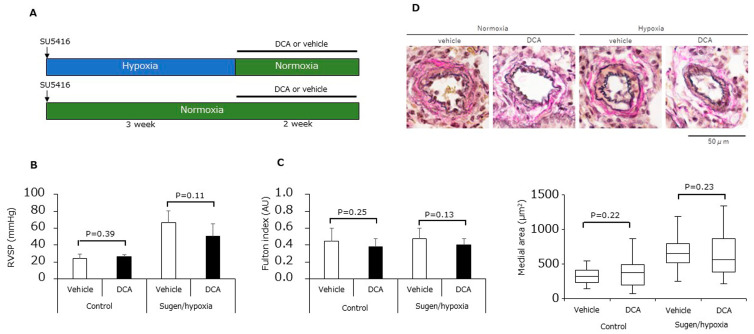
Effects of dichloroacetate in SuHx rats. (**A**) Experimental protocol. (**B**) Right ventricular systolic pressure (n = 6) for animals treated with either DCA or vehicle under either normoxia or hypoxia. (**C**) Fulton index (n = 6) for the animals in (**B**). (**D**) Medial area of peripheral pulmonary arteries for the animals in (**B**) (n = 6).

Finally, we assessed the effects of DCA in an animal model of PAH. Animal study protocol is shown in Figure 6A. Administration of DCA caused no significant differences to the right ventricle systolic pressure (RVSP) (Figure 6B), the Fulton index (Figure 6C), nor the medial area of peripheral pulmonary arteries (Figure 6D).

## 4. Discussion

To our knowledge, this is the first study evaluating the energy metabolism of PAH-PASMCs and non-PAH-PASMCs under normoxic and hypoxic conditions.

In previous studies on the energy metabolism phenotypes of PAH, PASMCs showed enhanced glycolysis, activation of the hypoxia-inducible factor HIF1 even under normoxia, and upregulation of oncogenes in human and animal models of PAH [13,14,15]. Reduced mitochondria-derived reactive oxygen species activate O_2_-sensitive HIF-prolyl-hydroxylases but directly facilitate HIF1αDNA binding and reduce the activity of p53 (which inhibits HIF1α at the levels of both transcriptional activity and stability). Thus, reduced mitochondrial activity can activate HIF1α independently of hypoxia. The decrease in pyruvate oxidation during the progression of PAH causes increased reliance on mitochondrial fatty acid oxidation to generate mitochondrial acetyl-CoA in order to support pulmonary vascular remodeling and PAH in mice [10]. Recently, novel factors were found to be involved in the metabolic pathway of PASMCs. Adenylate kinase 4 is a metabolic regulator in PASMCs interacting with HIF-1 and Akt signaling pathways to drive the pro-proliferative and glycolytic phenotype of PAH [16]. The glycolytic enzyme α-enolase-AMPK-Akt pathway regulates the pathogenic metabolic reprogramming observed in PASMC during PH [17]. Ras association domain family 1A-HIF-1α forms a feedforward loop driving hypoxia signaling in PH and cancer [18]. Here, we show that PAH-PASMCs undergo a metabolic shift toward glycolysis under normoxic conditions and that mitochondrial respiration is preserved under hypoxic conditions.

Understanding why PAH-PASMCs have differential features under normoxia and hypoxia is a complex question. In the 1920s, Warburg et al. showed that cancer cells produce lactic acid from glucose even under normoxia—known as the Warburg effect. The authors concluded that inhibition of mitochondrial respiration in cancer cells caused them to undergo a metabolic shift toward glycolysis. However, this idea has been recently reexamined, in part because many cancers exhibit the Warburg effect while retaining levels of mitochondrial respiration. Thus, in theory, cancer cells could carry out aerobic glycolysis and mitochondrial respiration concurrently [19]. Cancer cells exhibit robust flux through glycolysis and TCA cycle metabolism as well as their branched pathways (glutamine pathway, lipid pathway, and nucleotide pathway). TCA cycle oncometabolites (succinate, fumarate, D-2-hydroxyglutarate, and α-ketoglutarate) control DNA and histone methylation by regulating α-ketoglutarate-dependent dioxygenases [8]. These characters maintain their malignant phenotype and support tumor progression. PAH-PASMCs show many similarities to cancer cells, such as excessive proliferation and resistance to apoptosis. Therefore, we hypothesize that PAH-PASMCs could carry out glycolysis under normoxia and mitochondrial respiration under hypoxia in the same manner as many cancer cells. The reason that mitochondrial respiration is preserved in PAH-PASMCs under hypoxic conditions could be the availability of alternative fuel sources. One possibility is that glutaminolysis generates α-ketoglutarate to preserve mitochondrial respiration [20]. Further investigation is required to fully elucidate the preservation of mitochondrial respiration under hypoxia in PAH-PASMCs.

Michelakis et al. reported that PAH-PASMCs showed mitochondrial dysfunction with suppressed expression and function of voltage-gated K+ channels, which leads to PASMCs becoming resistant to apoptosis and the remodeling of pulmonary arteries [21,22]. This study also showed that treatment with DCA partially ameliorated or completely reversed pulmonary hypertension in animal models of PAH. Moreover, DCA treatment resulted in an impressive improvement in mean pulmonary arterial pressures for a small number of PAH patients [23]. Sutendra et al. reported that PDH is inhibited by hypoxia-inducible factors and tumor necrosis factor TNFα in PAH-PASMCs, which decreases the rate of conversion of pyruvate into acetyl-CoA in the mitochondrial matrix [24]. Evidence for glycolysis contributing to the development of PAH comes in part from the observation that pharmacologic or genetic inhibition of the positive glycolytic regulator PFKFB3 (6-phosphofructo-2-kinase/fructose-2,6-bisphosphatase 3) ameliorates the development of pulmonary hypertension in animal models [25]. The differences between the data we present here and these previous findings could be explained by the fact that we observed a shift toward glycolytic metabolism in PAH-PASMCs under normoxic conditions. However, treatment with DCA did not improve the symptoms of PAH in our study, in contrast to the results reported by Michelakis et al. This could be explained by DCA treatment being effective under normoxic conditions but not hypoxic conditions because peripheral pulmonary arteries are typically under hypoxic conditions. Moreover, to our knowledge, there have been no further studies using DCA for PAH treatment in humans or clinical trials reported.

The widely used drug metformin acts to inhibit mitochondrial complex I, thus decreasing the TCA-cycle intermediates necessary for tumor growth [26]. Indeed, pharmacologic inhibition of glutaminase 1, which is known to reduce mitochondrial respiration, attenuated rodent PAH [20]. Therefore, we believe that inhibition of mitochondrial respiration may be a promising therapy for PAH.

### Limitation

This study had some limitations. Normalization of the lactate amount and the data from Cell Mito Stress test and Glycolysis Stress test is recommended because chronic hypoxia increased the proliferation of PASMCs, which might affect the results of the present study. GLUT1 and GLUT4, which are known as glucose transporters, are important for cellular uptake of glucose. Further investigation is needed on the role of GLUT1 and GLUT4 in PAH-PASMCs under normoxia and hypoxia. Energy metabolism is complex, and a lot of enzymes except for PDK1, PDHA1, and LDHA are involved in energy metabolism. Investigations on other enzymes, metabolites, and metabolome analyses would be warranted to analyze the detail of energy metabolism in PAH-PASMCs. Furthermore, investigation of the differences in response to O_2_ concentration would contribute to elucidating the mechanism. We did not investigate the specific/selective drugs that inhibit mitochondrial complex I. Further study is needed to examine the drugs that inhibit mitochondrial complex I, which would be novel therapeutic drugs for PAH.

## 5. Conclusions

This study showed evidence for a hypoxia-induced shift in ATP production from glycolysis to mitochondrial respiration in PASMCs in PAH. This state of metabolic balance may be important for the pathogenic characteristics of PAH-PASMCs, such as excessive proliferation and resistance to apoptosis and hypoxia. Therefore, targeting mitochondrial respiration in PAH-PASMCs under hypoxic conditions may be a novel therapeutic strategy to treat PAH.

## Figures and Tables

**Table 1:** Patient characteristics of PAH-PASMCs.

Patient	Gender	Age
1	F	13
2	F	18
3	F	30
4	F	29
5	M	18
6	M	6

F: female, M: male.

**Table 2:** Primer sequence for real-time PCR.

Gene Symbol		Sequence
*PDK1*	F	CATGTCACGCTGGGTAATGAGG
	R	CTCAACACGAGGTCTTGGTGCA
*PDHA1*	F	GGATGGTGAACAGCAATCTTGCC
	R	TCGCTGGAGTAGATGTGGTAGC
*LDHA*	F	GGACAAGTTGGTATGGCGTGTG
	R	AAGCTCCCATGCTGCAGATCCA
*ACTB*	F	CACCATTGGCAATGAGCGGTTC
	R	AGGTCTTTGCGGATGTCCACGT

F: forward primer, R: reverse primer.

**Table 3:** Basal OCR, ECAR, and ATP production in PAH-PASMCs and non-PAH-PASMCs under normoxia and hypoxia.

	Normoxia			Hypoxia		
	Non-PAH-PASMCs	PAH-PASMCs	*p* Value	Non-PAH-PASMCs	PAH-PASMCs	*p* Value
Basal OCR (pmol/min)	79 ± 4	70 ± 5	<0.01	22 ± 3	39 ± 4	<0.01
Basal ECAR (mpH/min)	37 ± 2	44 ± 3	<0.01	32 ± 3	44 ± 3	<0.01
ATP production (pmol/min)	70 ± 2	59 ± 2	<0.01	14 ± 2	31 ± 3	<0.01
Maximal respiration	372 ± 49	387 ± 31	0.16	68 ± 26	176 ± 34	<0.01
Spare respiratory capacity	293 ± 46	317 ± 27	<0.01	46 ± 25	136 ± 28	<0.01
Coupling efficiency	0.89 ± 0.04	0.85 ± 0.07	<0.01	0.65 ± 0.04	0.81 ± 0.10	<0.01

OCR: cellular oxygen consumption rate; ECAR: extracellular acidification rate; ATP: adenosine triphosphate.

**Table 4:** Glycolytic functions of PAH-PASMCs and non-PAH-PASMCs under normoxia and hypoxia using the Glycolysis Stress test.

	Normoxia			Hypoxia		
ECAR (mpH/min)	Non-PAH-PASMCs	PAH-PASMCs	*p* Value	Non-PAH-PASMCs	PAH-PASMCs	*p* Value
Glycolysis yield	21 ± 10	42 ± 8	<0.01	14 ± 4	28 ± 6	<0.01
Glycolytic capacity	11 ± 7	15 ± 3	<0.01	7 ± 1	15 ± 2	<0.01
Glycolytic reserve	10 ± 4	27 ± 5	<0.01	6 ± 3	13 ± 5	<0.01

ECAR: extracellular acidification rate.

## Data Availability

The datasets generated during the present study are available from the corresponding author upon reasonable request.
